# International Dental Federation and International Commission of Education: Second General Meeting, Held at Stockholm, Sweden, 1902

**Published:** 1902-11

**Authors:** 


### INTERNATIONAL DENTAL FEDERATION AND INTERNATIONAL COMMISSION OF EDUCATION: SECOND GENERAL MEETING, HELD AT STOCKHOLM, SWEDEN, 1902.

### INTERNATIONAL DENTAL FEDERATION.

*Wednesday, August 19, 1902.*

THE opening session of the second general meeting of the International Dental Federation was held in the Caroline Institute, Wednesday, August 19, 1902, with Professor Lindstrom in the chair.

Professor Lindstrom, in fitting words, welcomed the members of the Federation.

Dr. Elof Förberg then addressed the Federation in behalf of the local committee, expressing in eloquent terms the satisfaction of the Swedish Dental Society in having the Federation meet at Stockholm.

After these preliminary proceedings, Professor Ch. Godon, Paris, delivered his presidential address, as follows:

### PRESIDENT'S ADDRESS.

MR. CHAIRMAN, LADIES AND GENTLEMEN,—As we are about to begin the sessions of the International Dental Federation, our thoughts are naturally carried back to our last and memorable meeting in the University of Cambridge, just a year ago,—a meeting the remembrance of which will never be erased from the minds of all who attended it. It seems to me that this meeting could not be more appropriately opened than by addressing our most cordial and respectful greetings to Sir Michael Foster, the eminent scholar who honored us last year by presiding over our gathering. It was at that session that we were invited by our friend and colleague, Dr. Förberg, in behalf of the Swedish Dental Society, to hold our present meeting in the city of Stockholm; and I can assure you that their hospitality is greatly appreciated by all of us. We should not forget that while our work has a scientific and professional purpose, it is also a task of peace and universal harmony, and under the auspices of what country other than Sweden could gatherings of this nature be more properly held? Sweden should feel a proud gratification in the reflection that the name of Dr. Alfred Nobel, the object of whose untiring efforts has been to promote ideas of peace and universal fraternity, stands out among the contingent of learned men that she has furnished to the world. All scientific and industrial associations, the number of which can never be in excess, uniting periodically upon the same spot men from countries different in language and habits for the purpose of discussing scientific topics for the better development of the different branches of human activity, must be counted among the number of institutions that have contributed in the largest degree to inculcating peace and fraternal sentiments among men, and therefore we could not do anything more fitting than to place this gathering under the tutelary patronage of that great human benefactor.

We are happy to express to the Swedish Dental Society our heartfelt thanks for their kind invitation and for the warm welcome they have tendered us, but to Professor Lindstrom are we especially indebted for consenting to honor our association by presiding over its meetings. We should feel complimented that, following Professor Gariel and Sir Michael Foster, who were the presiding officers at the organization of the Federation and at its first meeting respectively, we should be able to-day to open our first session with Professor Lindstrom in the chair, and that under these auspices the Federation should step forward in the path assigned to it.

Our sessions beginning under auspices of this nature cannot fail to be successful. Delegates are present in considerable number, and the questions to be discussed are numerous. We are glad to find among the delegates the majority of those who took part in our last year's discussions, and no other body of men could be better qualified to treat of the general questions that are of foremost importance to the future of dentistry. Others have come to join our force; to them we extend our most cordial thanks. We wish also to express our appreciation to the members of the American Dental Society of Europe, as they will have a considerable share in increasing the importance of the session, first, because of the part they will take in its work, and also because they have consented to hold joint sessions with us and with the Swedish Dental Society,—reunions which will make of this week a memorable one, as during this extent of time it will undoubtedly be possible to treat of questions belonging to all the branches of our special calling. We also thank the authors of the different communications for the earnestness with which they have treated the questions they undertook to study, and their reports will serve as a basis to the work of the present session.

Last year, at Cambridge, we had before us the completion of our organization, the adoption of a constitution, the appointment of the necessary committees, and the planning of our future line of work, as it was necessary to define the field of action of the Federation; also, to reassure all those who feared any interference on our part with national affairs; in one word, to limit the field of activity in which we could exercise our influence without fear of wounding legitimate susceptibility. Each country depends on its habits, historical evolution, and laws, and, as all these conditions differ greatly, our International Commission cannot pretend—as our colleague, Professor Hesse, of Leipzig, has stated—to do anything beyond expressing opinions and giving advices which have no real authority, but which have, however, a certain moral influence because of the standing of the members of the Federation, and may therefore be taken into consideration by those qualified in each country to voice decisions of legal power. Your functions are hence limited to that of a great international advisory council on dentistry. The discussions have been kept within the bounds of theory and philosophic generalities, and there is no cause for any one to complain that it should have been thus, especially when we recall Sir Michael Foster's remarkable address, in which he pointed out the most important requirements of the different kinds of preliminary education preparatory to the study of the various professions, that of dentistry in particular. He said that education should be fashioned after the manner of a cone, starting from a broad base and narrowing to an apex, for it is the conical bullet that has penetrating power. For each profession the cone should be different, should be fashioned in different ways, though in each case it should start from the same broad base. He indicated what this cone should comprise, analyzing the essential requirements for the dentist.

This admirable address was followed by one from an eminent surgeon of the same university, in which he defined, with as much authority as the previous speaker, the differences which should exist between the trainings of the physician, the surgeon, and the dentist, and in concluding affirmed the necessity for the dentist to be especially trained from the beginning to the end of his course of professional studies, as had been previously asserted by Dr. Kirk, the dean of the faculty of dentistry of the University of Pennsylvania, and Dr. Brophy, the president of the International Commission of Education. Dr. Kirk insisted upon the importance of undertaking the manual education early in life, for a period quickly arrives in later years when such training becomes impossible. It was this fact which was already recognized by Sir John Tomes, the Nestor of dental education in England, as Dr. Kirk has called him.

Some members, Dr. Arkövy, of Budapest, among them, maintain altogether opposite ideas,—namely, that dentistry is a medical specialty similar to all other specialties of the healing art, and must therefore be taught under the same conditions as these specialties are. They claim that the preliminary education of the dentist should be of a purely medical character, that the candidate should be a holder of the medical degree, and that only then should he take a special course in dentistry. Others advance opinions favoring a mixed education taken from both the medical and dental curricula.

These theoretical discussions have given rise to three questions which the members of the Commission have been asked to treat. Some national federations have also been requested to discuss these topics, and it remains with the Commission of Education to draw from these discussions clear and precise statements that shall serve as a basis to our discussions, and if possible to bring about a general understanding among our delegates. But the object of the Federation is not limited to the study of the best system of dental education; public dental hygiene is just as important a reason for its existence, and it is this topic that presents the greater degree of interest to the public and to the various governments. The Commission appointed last year has prepared reports in which they devote especial attention to the present status of public dental hygiene in the various countries here represented. These reports have awakened an interest on this important subject, and new members have asked to contribute towards solving this problem of unquestionable value and importance. The Commission will have to outline its plan of work for the future by determining the questions to be examined and discussed in the following meetings by the aid of suitable reports, as has been done already in the case of the Commission of Education.

There is another question on which a report has been prepared.

It refers to the federation of dental schools. A proposition embodying this idea was presented at the Third International Dental Congress, where it was seriously considered. As a matter of fact, it exists already as a national body in the United States, and comprises more than two-thirds of the American schools. It has helped considerably towards the unification of the dental curriculum, and is a factor in the progress of dental education in that country. In France a similar federation is being organized. We all realize what a mighty influence such an international body would have over the great problem of dental education, if, while respecting the autonomy of national federations, it were possible for it to bring about the acceptance by all dental schools the world over of a uniform programme comprising a minimum number of studies embracing all the topics a dentist should be familiar with in order to deserve this qualification, and also in order to be able to practise his profession to the benefit of humankind. It is to be hoped that the discussions which will undoubtedly follow the voluminous report at hand will bring about the realization of this plan.

The work of the Federation during the past year calls for the appointment of new committees. We must also set right the basis of the Federation, which is formed by the different national federations, which unfortunately have not been organized as yet in a regular way.

Lastly, we must see to it that the different governments take an interest in the work of the Federation, appointing delegates to our annual sessions, so that they may be able to learn from official sources the resolutions which we may adopt and which could be taken into consideration for the enactment of new legislative measures.

Working thus, we will advance towards the Fourth International Dental Congress, the preparation of which has been intrusted to us. It is there that the dental world will be enabled to make a new inventory of the recent progress realized by odontological science.

If we are able during this quinquennial period to insure that the work of the Federation will be carried on regularly, if we can carry it to altitudes where ideas shall meet in peaceful controversies, to unite later on and collapse as the clouds do after a most terrific storm producing copious and fruitful rains, which finally result in abundant crops, then we may be able to retire with quiet consciences, leaving the future to others, feeling that we have not wasted our time and that our efforts have been fraught with beneficial results.

Dr. Florestan Aguilar, the treasurer, then presented his report.

Addresses followed by Dr. Hesse, Germany; Dr. Harlan, United States; Dr. Harding, England; Dr. Franck, Austria-Hungary; Dr. Heidé, France; Dr. Guerini, Italy; Dr. Guldeberg, Norway; Dr. Frick, Switzerland; Dr. Morrisson, Australia; Dr. Royce, the American Dental Society of Europe; Dr. Weber, Finland.

